# Supplementary material for: Are carbon emissions trading and green financial instruments synergistic? -Comprehensive quantitative research based on content analysis
Source: PLoS One. 2024 Mar 7;19(3):e0298601. doi: 10.1371/journal.pone.0298601 (PMC10919601; doi:10.1371/journal.pone.0298601)
Supplement: S1 Data — (ZIP) [file pone.0298601.s001.zip › Data/地级市Z_F工作报告.zip!/░▓╗╒/┴∙░▓╩╨/└┤╫╘╣1⁄2╓┌║┼í╛┬φ┐╦╩2╛▌═°í┐_2010.docx]

各位代表：
　　现在，我代表市人民政府，向大会作政府工作报告，请予审议，并请市政协委员和其他列席人员提出意见。

一、团结拼搏，五年发展跨上新台阶

　　过去的五年是不平凡的五年，是在首届政府转体制、打基础之后，实现加快速度、提升水平的五年。五年来，全市人民在中共六安市委的坚强领导下，深入学习实践科学发展观，团结一致，开拓奋进，圆满完成了市二届人大一次会议提出的各项目标任务，六安迈入了加快发展的新阶段。
　　这五年，是经济持续快速发展、综合实力明显增强的五年。全市生产总值由278.5亿元增至584亿元，年均增长13.7%，总量由全省第10位上升至第6位。财政收入由16.1亿元增至52.1亿元，年均增长26.5%。工业主导地位显现，三次产业结构由31.6:34.7:33.7调整为23.7:40.7:35.6，工业对经济增长贡献率达到58.1%；规模工业增加值由34.6亿元增至187.1亿元，总量由全省第12位上升至第9位，增幅保持全省前列；高新技术产业产值由5亿元增至62亿元。农业基础地位得到加强，粮食总产连续6年创历史新高，霍邱县连续5年夺得全国粮食生产先进县标兵，寿县第4次荣获全国粮食生产先进县。国家级、省级和市级农业产业化龙头企业分别达到2家、35家和160家。社会消费品零售总额由99.7亿元增至235.5亿元，年均增长18.8%。旅游业方兴未艾，建成4A级景区8个。
　　这五年，是投入力度不断加大、城乡面貌深刻变化的五年。累计完成固定资产投资1062.9亿元，是前5年的4.9倍。合武铁路、合六叶、合淮阜、六潜、合武高速公路六安段，华电六安电厂一期、皋城500千伏输变电、临淮岗洪水控制工程、白莲崖水库等一批重大项目建成运行。中心城市新一轮总体规划修编完成，各项工程累计完成政府性投资近75亿元，淠河橡胶坝等170项重点工程相继完工，建成区面积由29.3平方公里扩展到55.3平方公里、人口由34万人增至60万人，公用设施配套逐步完善，管理水平进一步提升，荣获中国人居环境范例奖、水环境治理优秀范例城市和安徽省园林城市等称号。县城和50个重点镇建设加快推进，城镇化率达到36.9%，五年提高近10个百分点。建成村村通公路6837公里，金寨县被评为全国农村公路建设示范工程先进单位；治淮5项骨干工程和重点地质灾害点治理全面完成。生态建设加快推进，全市森林覆盖率36%，舒城县被授予全国绿化模范县，霍山县成为中西部地区第一个国家级生态县。
　　这五年，是各项改革逐步深化、对外开放成效明显的五年。国有和集体企业改革基本完成，农村综合、财政、卫生、集体林权、水管等体制改革和事业单位用人制度改革积极推进，事业单位岗位设置管理工作全面启动，工商系统市场管办分离改革顺利完成。行政审批制度改革不断深化，取消和调整审批项目82项，政务中心服务水平明显提高。金融改革进一步深化，金融生态环境明显改善，累计新增贷款249亿元，是前五年的近6倍。对外开放不断扩大，累计到位内资407.4亿元、外资4.3亿美元，分别是前五年的4.5倍和3.3倍；进出口总额由1.13亿美元增至3.3亿美元。全民创业行动充满活力，非公有制经济比重达到60%以上。
　　这五年，是社会事业全面发展、人民生活显著改善的五年。城镇居民人均可支配收入由6103元增至13001元；农民人均纯收入由2117元增至4010元。自2007年实施民生工程以来，累计投入56亿元，惠及650万城乡居民，连续三年获全省一等奖。市人力资源市场、社会保障服务中心和科技创业中心建成使用。科技特派员创业行动获全国先进，霍山、舒城、金寨、霍邱四县被授予全国科技进步县称号。义务教育经费保障机制改革深入实施，中小学D类危房改造任务全面完成，高中教育普及率达76.5%。城乡医疗服务能力和水平显著提高，新型农村合作医疗参合率达94.6%，皖西卫生职业学院申报取得重大进展。就业和社会保障体系进一步健全，城镇新增就业18.6万人，城镇登记失业率控制在3.85%以内。五大保险参保人数由89.6万人次增加到205.9万人次，连续六年提高企业退休人员养老金水平。城镇低保、农村低保均实现应保尽保。大力实施整村推进、产业扶贫、培训转移和社会帮扶，贫困人口减少至42.6万人。低生育水平保持稳定，计划生育保障体系和工作机制逐步完善，以人为本的计生优质服务工作深入推进。文化、广电、体育、新闻出版业健康发展，市老年活动中心、青少年活动中心和全民健身活动中心相继建成使用，人民群众精神文化生活日益丰富。寿县荣获全国文化先进县和全国文物工作先进县称号。统计、档案、保密、地方志、地震、气象、对台、外事侨务、民族宗教、人防、妇女儿童、老龄和残疾人等各项事业都取得了新的成绩。
　　这五年，是和谐社会建设扎实推进、政府自身建设进一步加强的五年。深入推进“平安六安”、社会治安防控体系和应急管理体系建设，加强食品药品安全监管，人民群众安全感和对社会治安满意度指数不断上升，霍山县力夺全国社会治安综合治理“长安杯”。安全生产形势平稳，事故总量、死亡人数等4项绝对指标全面下降。支持驻地部队建设，国防动员和民兵预备役工作扎实开展，首次荣获“全国双拥模范城”称号。坚持依法行政，全面完成市本级行政执法依据清理和规范行政处罚自由裁量权工作，开展城市管理相对集中行政处罚权和综合行政执法试点，“五五”普法扎实推进。自觉接受人大法律监督、政协民主监督和社会舆论监督，共办理人大议案15件、代表建议441件和政协委员提案1108件，办结率100%。推进科学民主决策，重大事项集体决策、专家咨询、社会公示和听证制度逐步完善。全面推行信息公开，扩大政务公开，市政府网站连续5年进入全国50强。建立并坚持信访接待日制度，认真落实信访责任制和问责制。加强勤政廉政建设，强化行政监察和审计监督，政风行风和机关效能建设取得明显成效。
　　过去的2009年，是新世纪以来经济社会发展最为困难的一年。一年来，我们认真贯彻中央和省各项政策措施，全力以赴保增长、保民生、保稳定，实现了经济社会平稳较快发展。一是抓工业保企业。深入开展“发展工业学霍山”活动，组织开展企业经验交流、工业观摩和现场会，营造了浓厚的工业发展氛围。扎实开展“企业帮扶年”活动，派出206个帮扶组、25名市级负责同志、719名机关干部进驻企业。制定并认真落实一系列保增长的政策措施，全市筹集6亿多元资金用于企业贷款贴息、投资引导、贷款风险补偿和中小企业投资担保机构增资，市工投公司开始运营。编制出台了《六安市工业重点产业发展规划》，推进钢铁双百亿工程，实现钢铁产值103亿元。新增规模企业132家，总数达到819家，亿元企业增至126家。二是抓项目争投入。抢抓扩大内需机遇，争取中央投资项目1002个、16.1亿元，居全省首位。投资26亿元的华电六安电厂二期、25亿元的100万吨球墨铸造、18亿元的500万吨李楼矿山采选等重大项目开工建设。全年完成固定资产投资355.2亿元，增长38.6%；其中3000万元以上重点项目完成投资222亿元。到位内资、外资分别达到193亿元、1.33亿美元，增长45.6%和20.4%。与8家省级银行机构签署了战略合作协议，组织大型银企对接会9场，全年新增贷款突破100亿元，银行业金融机构盈利7.65亿元。三是抓农业稳基础。加强耕地保护，扎实推进粮食双百亿工程，粮食总产突破90亿斤大关，加工产值突破80亿元；农业产业化倍增计划加快推进，全年农产品加工产值227.9亿元，增长26%。积极发挥十大特色农业产业协会的组织引领作用，开展了“神农杯”创业竞赛活动。无公害、绿色和有机“三品”认证总数达180个。四是抓城建强平台。15亿元城投债券成功发行，启动淠河城区段12公里和1.4平方公里月亮岛综合治理，开启了中心城市建设新篇章；提前组织新一轮城市总规和城市防洪规划修编，实施60万平方米中央森林公园、淠干延伸整治等重大项目，53项重点工程基本建成，完成政府性投资20.3亿元。各县城和小城镇建设亮点纷呈。五是抓流通活消费。启动商贸流通双百工程，沃尔玛、时代购物中心等一批境内外知名企业落户六安并正式开业。积极推进家电、汽车、电脑等下乡活动，全年发放补贴突破1亿元，农村消费增幅首次高于城市。旅游总体规划通过评审，红色旅游“139”计划顺利完成，全年共接待游客577.6万人次，创旅游总收入31.6亿元。力保对外贸易，全年完成出口3.21亿美元，发展情况好于全省。六是抓民生促和谐。全面实施28项民生工程，投入资金33.7亿元，不断创新工作机制，在全省实现“五个率先”。全市25.6万名困难农民有了最低生活保障，61.4万城镇居民参加医保，6.4万城乡困难群众获得医疗救助，536.6万农民参加新农合。农村五保户集中供养率比上年提高10个百分点。各项社会事业全面发展，社会和谐稳定。
　　各位代表！五年成绩来之不易，辉煌历程充满艰辛。这是省委、省政府和市委正确领导的结果，是全市各族人民团结奋斗、顽强拼搏的结果，离不开市人大、市政协和社会方方面面的关心爱护、监督支持，离不开历届地委、行署和市委、市政府打下的良好基础。在此，我代表市人民政府，向全市人民，向广大工人、农民、知识分子、干部、驻地官兵和政法干警，向各民主党派、各人民团体和社会各界人士，向所有关心支持六安现代化建设的朋友和在我市创业的投资者、建设者表示崇高的敬意和衷心的感谢!
　　在肯定成绩的同时，我们也清醒地认识到，六安虽然在全省经济发展大局中增速趋前，但总量居中、人均靠后的格局尚未改变；工业化水平较低，结构不优，发展后劲有待增强；农业基础仍比较薄弱，农业产业化水平较低；城镇化水平不高，制约城乡和区域协调发展的矛盾仍很突出；城乡居民收入水平不高，改善民生的任务十分艰巨。政府工作中还存在一些缺点和不足，主要是思想解放不够，行政执行力、落实力有待进一步提高，机关作风需要进一步改进等。对此，我们一定高度重视，采取有效措施加以解决。

二、开拓进取，推动科学发展新跨越

　　今后五年，是我们抢抓战略机遇、加速六安跨越、全面建设小康的关键时期。站在新的历史起点，面对新的机遇和挑战，我们必须增强时不我待的责任感和紧迫感，利用一切有利条件，调动一切积极因素，致力发展，加快发展，科学发展，逐步缩小与全国发展的差距，赶上全省发展的步伐，奋力开创六安经济社会发展新局面。
　　根据市委二届十四次全会精神，今后五年政府工作的总体要求是：高举中国特色社会主义伟大旗帜，深入贯彻落实科学发展观，以推动跨越发展为主导，以提高综合实力为主题，以壮大主导产业为主线，以改善民生为主旨，抢抓机遇、增创优势，深化改革、扩大开放，强力推进工业化核心战略、招商引资主战略和城镇化带动战略，全面提升工业、农业、中心城市、商贸流通四大双百工程，全力推动经济社会发展迈大步、上台阶，奋力实现向农业强市、工业大市、财政富市的跨越。
　　今后五年政府工作的主要目标是：
　　——综合实力再上新台阶。到2014年，全市生产总值达到1200亿元左右，较2009年翻一番，年均增长13%以上。全社会固定资产投资超过1000亿元，年均增长20%以上。社会消费品零售总额逼近500亿元，较2009年翻一番，年均增长16%以上。城镇居民人均可支配收入、农民人均纯收入分别突破23000元和6200元，年均增长10%和9%以上，达到全省平均水平。
　　——推进向农业强市跨越。到2014年，粮食总产稳定在100亿斤以上，农产品加工产值达到550亿元，年均增长20%以上；培育年销售收入超30亿元龙头企业1-2家，超亿元100家以上。
　　——推进向工业大市跨越。到2014年，规模工业增加值突破400亿元，较2009年翻一番，年均增长20%以上；培育年产值超百亿元产业3个，年产值超百亿元企业3家、超10亿元30家，亿元以上企业达200家以上，规模以上企业1500家以上。
　　——推进向财政富市跨越。到2014年，财政收入突破130亿元，较2009年翻一番多，年均增长20%以上，占生产总值比重达到11%。
　　——社会事业取得新发展。到2014年，文化产业增加值占生产总值的比重达到4%以上，初步建成覆盖城乡的公共文化服务体系；五年新增城镇就业12万人，城镇登记失业率控制在4.2%以内，社会保障体系更加健全；基本普及高中教育，基本医疗保障实现全覆盖，社会治安和安全生产状况进一步好转，人口自然增长率控制在7.6‰以内。
　　实现上述目标，今后五年工作的主要任务是：
　　第一，着力壮大支柱产业。加快实施工业重点产业发展规划，围绕汽车零部件、钢铁、机电、农产品深加工、建材、电力、纺织服装、特色高新技术、物流业等9大主导产业，推进产业纵向延伸和横向配套。深入实施农业产业化倍增计划，全面推进优质粮食、畜禽、水产、茶叶、油茶等主导农产品基地建设，大力发展特色农产品精深加工，做大做强龙头企业，初步构建具有六安特色的农业产业体系。深入实施旅游总体规划，打造全国知名旅游目的地。着力壮大金融服务业，积极构建地方性金融体系。有序发展房地产业，扩大房地产有效需求。
　　第二，着力转变发展方式。积极开展质量兴市活动，大力实施名牌战略和技术标准战略，推动自主创新，发展一批高新技术企业和产品，实现高新技术产业产值150亿元以上。加强产学研合作，搭建中小企业公共技术创新平台，实现专利授权量年均增长20%以上。推进集约节约发展，抓好重点行业和重点企业节能降耗，发展循环经济、低碳经济，实现主要污染物减排目标。大力培育新兴产业，围绕新能源、新材料、节能环保、电子信息等重点产业，实施技术创新工程。强化产业集聚，实现9个省级开发园区和工业集中区工业增加值占全市工业增加值比重达到80%以上。加强林业重点生态工程建设，新增人工造林50万亩，森林覆盖率提高2个百分点。
　　第三，着力统筹城乡发展。加快实施中心城市新一轮总体规划，实现建成区面积达80平方公里、人口80万人。强力推进淠河综合治理，建设功能齐全、配套完善、生态宜居的滨水新区。围绕打造全省一流的生态文明城市目标，提高城市品位，改善城市环境，理顺管理体制，提高管理水平，确保2011年成功创建国家园林城市。统筹城镇化进程，突出抓好5个县城、叶集试验区和50个重点镇建设。促进区域均衡发展，推进扩权强镇工作，增强县域经济自主发展活力。加快新农村建设，突出抓好一批示范点，促进城镇化与新农村建设良性互动。深入推进农村土地承包经营权流转，支持多种经营主体发展适度规模经营。围绕综合交通枢纽体系建设，建成周六高速，开工建设济祁高速寿县—金安—舒城段等一批重大交通项目，新改建农村公路3000公里以上。谋划实施新一轮治淮工程，开展沿淮洼地和中小河流治理，推进病险水库除险加固和农村人口饮水安全工程建设。加快“数字六安”进程，完善公众信息服务、政务服务等平台建设。
　　第四，着力深化改革开放。深化农村综合改革，全面完成乡镇机构、农村义务教育和县乡财政管理体制改革。继续推进集体林权制度改革，建立健全支持林业发展的各项制度。健全农村市场体系和农业社会化服务体系，积极推进新农村现代流通服务网络工程建设。深化投融资体制改革，积极发展资本、土地、劳动力和技术等要素市场。大力推进企业上市和境外融资，力争5家企业上市。以皖江城市带承接产业转移示范区规划实施为契机，进一步扩大对外开放，坚持大招商、招大商，引进一批附加值高、牵动性强的重大项目。推进合六工业走廊和新桥国际产业园、舒三工业集聚区建设，全力打造承接长三角区域产业转移的重要平台。加快融入合肥经济圈，编制实施我市基础设施、产业布局等重点专项规划，建设城际快速通道，推进大别山优质水源供应工程。扩大对外贸易，实现进出口总额年均增长12%以上。
　　第五，着力构建文化强市。以社会主义核心价值体系为根本，加强社会公德、职业道德、家庭美德、个人品德和未成年人思想道德建设，提高全民素质。紧紧围绕创建省级文明城市目标，加大创建工作力度，突出抓好净化、美化、绿化、亮化工程。着力构建公共科技文化服务体系，规划建设市科技馆、体育馆和报业大厦，建成市图书馆、文化馆和新皖西大戏院。坚持面向基层、服务群众，完成乡镇综合文化站和村级农家书屋建设任务，加快公共文化向基层和农村延伸。繁荣文学艺术创作，进一步加强对物质和非物质文化遗产的挖掘和保护。深入实施文化品牌带动战略，弘扬皋陶文化、楚汉文化、红色文化等品牌，打造一批文化精品。加快文化产业发展，规划建设2-3个省级文化产业园区、8-10个市级文化产业园区，培育引进具有较强竞争力的文化龙头企业。深化文化体制改革，健全文化市场监管长效机制。
　　第六，着力促进社会和谐。优先发展教育事业，促进义务教育均衡发展，大力发展职业教育，进一步重视和支持幼儿教育、高等教育。优化城乡中小学布局，加快学校标准化建设，全面完成中小学校舍安全工程。深化医药卫生体制改革，建设覆盖城乡居民的公共卫生服务、医疗服务体系。坚持以创业带动就业、以培训促进就业，健全统筹城乡的就业服务体系，扩大就业规模，改善就业结构。建立健全覆盖城乡居民的社会保障体系，完善被征地农民基本生活保障制度，推进新型农村养老保险和城镇老人居家养老服务试点，着力解决低收入群众住房困难。推进全民健身长廊工程建设，广泛开展全民健身运动，提高竞技体育水平。稳定低生育水平，综合治理出生人口性别比，提高出生人口素质，统筹解决人口问题。加强“平安六安”建设，强化安全监管，完善应急体系和社会治安防控体系，确保社会和谐稳定。

三、真抓实干，开创今年工作新局面

　　2010年是新一届政府任期的第一年，也是“十一五”规划的收官之年，做好今年的经济社会发展工作具有十分重要的意义。从宏观形势看，国家将继续实施积极的财政政策和适度宽松的货币政策，特别是中部崛起规划和皖江城市带承接产业转移示范区规划的实施，为我们持续快速发展提供了十分有利的机遇。从我市来看，经济实力逐步增强，投资力度不断加大，发展条件明显改善，为我们加速跨越提供了有力支撑。但当前国际金融危机余波未尽，发展的复杂性不容低估。我们必须积极研究和把握宏观环境的新变化，在区域合作中抢占发展先机，在调整结构中培育主导产业，在竞相发展中增创后发优势，全面完成“十一五”目标任务，为顺利启动“十二五”、推进“三大跨越”奠定坚实基础。
　　今年经济社会发展的主要预期目标是：全市生产总值增长13%，财政收入增长18%以上，固定资产投资增长30%以上，规模工业增加值增长20%以上，社会消费品零售总额增长17%以上，城镇居民人均可支配收入增长10%以上，农民人均纯收入增长9%以上，城镇新增就业2.64万人，城镇登记失业率控制在4.2%以内，人口自然增长率控制在7.6‰以内，单位生产总值能耗完成“十一五”规划目标，化学需氧量和二氧化硫排放量分别下降5.44%和1.4%。实现上述目标任务，必须着力抓好以下八个方面工作：
　　（一）千方百计加大投入，增强经济发展后劲
　　推进项目建设。紧紧围绕国家投资方向，以承接产业转移为抓手，继续做好项目争取工作。积极谋划金寨钼矿开发、国电舒城电厂、大别山优质水源供应等一批重大项目，提升项目前期工作水平。健全和创新项目工作机制，全面落实规划、用地、环评等项目建设条件。尽快开工建设与首钢合作的钢铁深加工、大别山现代中药材工业园、金领欢乐世界等项目，加快实施华电六安电厂二期、霍邱铁矿采选、应流高新技术铸造基地、宝利嘉纺织、蓝翔玻业、新成铁薄板、财富汽车和杭埠粮食产业园、丽军军需产业园等一批重点在建项目，确保3000万元以上项目完成投资265亿元。
　　加快基础设施建设。牢牢把握国家扩内需重大机遇，突出抓好交通、水利、通讯等一批重大基础设施工程建设。争取尽快实施312国道六安段、206国道舒城段改造升级，支持周六高速、阜六铁路六安段建设，争取宁西铁路复线六安段开工，积极做好六安至安庆铁路、合六城际轻轨和济祁高速六安段前期工作。编制和完善水利规划，启动新一轮治淮工程，加快推进淠河、史河等主要内河整治、淠史杭灌区续建配套和大中型排灌泵站更新改造工程，力争实施寿西湖行洪区调整改造、城西湖蓄洪区治理工程，完成45座中小型病险水库除险加固工程。进一步加大对开发区、矿区和农村地区电网建设投资力度，推进配电网改造升级。加快推进3G网络无线城市建设步伐，实现“村村通宽带”目标。
　　加强金融工作。引导金融机构深入开展“六安发展我发展，我为六安做贡献”活动，强力推进全市活力金融体系建设，确保信贷投放持续稳定增长，力争贷款增速高于全省平均水平。加大银企对接力度，完善政银企合作机制。加快推进中小企业信用评级，完善中小企业贷款风险补偿各项政策，切实缓解中小企业融资难。加快市外金融机构引进步伐，支持设立交通银行六安分行，鼓励增设新型农村金融机构，积极搭建新型投融资平台，推进金融可持续发展。
　　（二）坚持工业化核心战略，加快新型工业化进程
　　做大工业总量。支持龙头企业通过联合、裂变等方式快速扩张，重点跟踪扶持星瑞齿轮、江淮电机、大昌矿业、应流集团、金安不锈钢、长江精工、迎驾集团、庆发集团、夏星食品、索伊电器、博微长安等一批主业突出、成长性好的骨干企业，努力培育一批拥有自主品牌和核心竞争力的大企业，力争年销售收入超亿元企业达150家，其中超10亿的企业突破10家，全年完成工业投资200亿元以上。鼓励中小企业围绕大企业开展协作配套，推动全民创业和回乡创业，力争全市规模企业接近1000家。继续深入开展“发展工业学霍山”活动，坚持“四个定期、五个倾斜”，全面抓好各项政策措施的落实，将应对危机帮扶制度转为领导干部驻企联络制度，实现帮扶工作常态化。加大培育支持力度，力争今年有1家企业成功上市，2-3家企业进入辅导期。
　　调整产业结构。采用新技术大力改造和提升传统产业，鼓励企业加大研发投入，重点推进应流超奥不锈钢、江淮电机超高效防爆电机和电动汽车电机、星瑞双离合自动变速器、世林LED照明、双龙机床和威玛叉车研发等技术创新项目建设。积极支持光伏、新型电光源、生物能源等新兴产业快速健康发展。落实科技创新政策，建设一批工程技术研究中心，增强企业技术创新能力。不断完善市科技创业中心孵化功能，进一步引导和扶持各类科技中介服务机构发展。加强公共技术检测平台建设，筹建省茶叶质量安全（皖西）检测中心，支持霍山茧丝绸产品质量监督检验中心争创国家级中心。加强创新人才队伍建设，注重培养一线技术创新人才。
　　强化产业集聚。按照集群式发展、产业链延伸、专业化分工的原则，加强9个省级开发园区规划、建设和管理，推进园区扩容升级，完善园区功能，强化融资、征地和用工保障，增强项目承载和集聚能力。六安开发区要力争完成基础设施投入4亿元以上，引进超千万元项目30个以上，亿元项目突破10个；各县区开发区要力争完成基础设施投入1.5亿元，引进超千万元项目20个以上，亿元项目突破5个。积极探索园区合作共建模式，吸引长三角、珠三角等地企业在我市建设园中园。采取多种措施，保障工业用地，提高土地资源利用效率。引导和促进乡镇工业集中区持续快速健康发展。
　　（三）大力发展现代农业，加速新农村建设步伐
　　发展现代农业。深入实施粮食双百亿工程，组织水稻产业提升和小麦高产攻关行动，力争粮食面积、单产刷新纪录，粮食总产冲刺95亿斤，力争粮食加工产值达到90亿元。支持中储粮六安油脂油料基地和粮源基地建设。推进土地流转机制创新，争取新增土地流转30万亩。积极推进农业标准化，加快县区现代农业示范小区和特色种植小区建设，力争新增标准化畜禽小区100个、水产养殖小区20个。加快实施农业产业化倍增计划，力争农产品加工产值达到255亿元，增长13%，亿元龙头企业发展到60家以上。重视发展农民专业合作组织，充分发挥十大产业协会作用，加快粮油、畜禽、水产、茶叶、油茶等产业基地建设。推进标准化生产，加强农业投入品生产、经营和使用管理。大力发展品牌农业，无公害、绿色和有机“三品”认证总数突破200个。
　　推进新农村建设。加快土地利用总体规划编制，严格落实耕地保护责任制。加强规划引导，整合项目和资金，全力抓好一批新农村示范点建设，着力改善村容村貌。加强农田水利基本建设和江淮分水岭综合治理，解决38万农村人口饮水安全。启动新一轮农村电网改造升级，继续实施新农村电气化建设。进一步深化农村公路养护体制改革，推进村村通延伸工程，新改建农村公路659公里。深化集体林权制度配套改革，加强林业生态建设，加快林业产业发展。巩固完善为民服务全程代理制，积极推进村级公益事业建设“一事一议”财政奖补试点，切实做好农村集体“三资”代理服务工作。
　　促进农民增收。认真落实各项支农惠农政策，加大扶贫开发力度，健全农民负担监管长效机制。整合培训资源，大力实施农村劳动力技能培训、新型农民科技培训、农村实用人才培养计划等工程，培训农村劳动力7万人。以发展农村二三产业为着力点，加快农民工创业园建设，继续开展“神农杯”创业竞赛活动。大力发展一村一品、一乡一业，扶持壮大农村特色产业和特色经济。加大农业支持保护力度，扩大农业保险覆盖面。
　　（四）突出抓好中心城市和县城建设，加快城镇化进程
　　加快中心城市重点工程建设。深入实施中心城市双百工程，完成重点工程三年行动计划，重点实施好淠河综合治理及滨河新区建设、淠干综合治理、中央森林公园、淠望路及淠史杭大桥、南门塔环境整治、城区路网和柔性路面改造、安置房建设、大雁河分段治理等63项新建续建工程，完成政府性投资26亿元。高度重视城市搬迁安置工作，确保群众利益，依法依规推进。进一步完善城市功能，注重路网管网建设和地下空间开发利用，加快老城改造和公厕、公交候车亭、健身休闲场地等配套设施建设。
　　提升中心城市品位。加快推进中心城市控制性详规编制，完成中心城市23个专项规划编制和“一岛两岸”城市设计。围绕建设国家园林城市目标，抓好梅山路、人民路等环境提升工程和皖西大道及东大门改造工程，确保城区绿化覆盖率达42%，人均公共绿地10平方米。改革城市管理体制，创新城市管理模式，充分发挥数字城管效能和作用，推进数字化、信息化管理。加大违法建设查处力度，开展市容环境热点问题专项治理，推进市容管理和环卫作业市场化改革。积极引导和促进住房与房地产业健康有序发展，提高住宅小区的建设品位和配套档次，加大廉租房、安置房等保障性住房的建设力度，提升物业管理水平。
　　推进县城和集镇建设。加强土地、城镇村规划编制实施和管理，实现城乡规划全覆盖。突出抓好5个县城和叶集试验区建设，着力拓展框架，完善功能，提高水平，形成特色。推进省扩权强镇试点，加快50个重点镇建设，积极有序引导人口、产业向县城和中心镇集中。
　　（五）大力发展第三产业，着力扩大消费需求
　　加快发展旅游业。加大重大旅游项目的谋划和旅游业发展三年行动计划实施力度，落实支持旅游业发展的政策和措施。加快推进寿州古城修复、金寨西庄温泉综合开发、霍山大别山主峰景区道路等一批旅游重大项目建设，加快天堂寨、万佛湖、八公山、独山苏维埃城等景区的转型升级，提升品位，提高市场竞争力。整合旅游资源，强化旅游宣传促销，健全完善旅游人才培训机制，启动全国优秀旅游城市目的地标准化建设。抓好大别山（六安）国家地质公园争创世界地质公园工作。加快推进生态游、乡村游、农家乐等特色旅游。
　　积极发展现代服务业。围绕构建与产业发展相适应的生产服务体系，大力发展现代物流、科技信息、通讯网络、商务金融等生产服务业。充分发挥六安交通枢纽优势，逐步建设一批现代物流园区，打造大别山和合肥经济圈区域物流中心。高度重视城市居民生活消费需求，积极发展汽车服务、社区服务、物业管理、养老和医疗保健等生活服务业，加快形成与城市功能相适应的服务业体系。扩大住房公积金覆盖面，充分发挥互助功能。
　　繁荣消费市场。加快实施商贸流通双百工程，推进城市商业网点规划布局实施，加强农村流通体系和服务网络建设，继续推进“万村千乡市场工程”和“新网工程”，加大供销系统为农服务力度，支持大型商业连锁企业向农村延伸网点。积极组织开展农产品产购销对接活动，认真落实家电、汽车等下乡及以旧换新政策措施。
　　（六）进一步扩大对外开放，激发经济发展活力
　　强力推进招商引资主战略。进一步改进招商引资考核、激励机制，整合招商资源，围绕重点区域，紧盯重点客商，突出重点产业，充分发挥招商小组、驻外联络处以及在外人才协会和商会作用，大力推进产业招商、专业招商、园区招商、以商招商和引凤还巢，积极动员和鼓励社会招商，确保到位内资、外资均增长15%以上。认真落实与央企对接合作成果，确保签约项目落地生根。
　　全方位开展区域合作。以实现“合六一体化”为目标，突出规划对接、产业合作、设施共建、资源共享、信息互通，努力把六安建成合肥经济圈加工制造业基地的核心区之一。积极参与泛长三角区域分工合作，发挥比较优势，整合优质资源，找准产业定位，培育主导产业。依托皖江城市带承接产业转移示范区，规划建设合六工业走廊和新桥国际产业园、舒三工业集聚区，形成呼应“一圈一带”发展的重要承接平台。认真落实省加快皖北**和沿淮部分市县发展**的政策措施，促进寿县、霍邱加快发展。
　　扩大对外经济贸易。落实稳定外需的各项促进政策，进一步挖掘进出口潜力，支持重点企业扩大外贸规模。转变外贸发展方式，优化出口商品结构，扩大进出口增长点，鼓励扶持新增备案企业做大做强进出口业务，积极承接发达地区外向型产业转移。实现进出口总额3.6亿美元，增长10%。争取合肥海关和省商检局尽快在我市设立分支机构。
　　（七）着力保障和改善民生，让人民群众得到更多实惠
　　拓展提升民生工程。把民生工程作为“民心工程”放在更加突出位置，围绕“学有所教、劳有所得、病有所医、老有所养、住有所居”目标，以实施33项民生工程为抓手，重点做好就业促进、扶贫解困、医疗卫生、百姓安居等各类直接关系民生的工作，全力解决群众最关心、最直接、最现实的利益问题。建立稳定多元的民生工程筹资机制，优化财政支出结构，确保新增财力向民生倾斜，全年投入民生工程资金不少于40亿元。巩固提高民生工程成果，坚持以政策宣传为引导、以制度创新为主线、以提高项目运营效率为目标、以健全协调推进机制为重点，强力推进各项民生工程工作的有效落实。
　　促进就业和再就业。全面落实税费减免和各项补贴政策，加大职业技能培训力度，加强劳动力供需衔接。积极鼓励自谋职业和自主创业，做好高校毕业生、转业退伍军人、被征地农民和残疾人就业，支持引导农民工回乡就业和创业；积极开展就业援助活动，重点促进零就业家庭就业和下岗失业人员再就业，实现再就业1.21万人，就业困难人员就业2600人。全面落实农民工政策，加强对用人单位执行劳动合同、工资分配、参加社会保险等的指导与监管，依法维护劳动者权益。
　　进一步完善社会保障体系。加强社保基金征缴和管理，扩大五大保险覆盖面，提高保障标准。贯彻落实养老保险关系转移接续办法，实施工伤保险市级统筹，着力抓好金寨、霍山全国新型农村社会养老保险试点。加强城乡社会救助体系建设，进一步提高城乡居民最低生活保障规范化管理和医疗救助水平，确保城镇未参保集体企业退休人员基本生活保障应保尽保。加快农村五保供养服务机构建设，重视老年人、残疾人和社会福利、慈善事业，做好防灾减灾和灾害救助工作。
　　（八）协调推进社会事业发展，维护社会和谐稳定
　　全面发展社会事业。加大文明创建力度，积极争创省级文明城市。加大对农村和薄弱学校的扶持力度，提升义务教育水平。实施新一轮中小学布局调整，加快城区义务教育阶段公办学校改扩建、市级职教园区和中小学校舍安全工程建设，支持引导规范民办教育健康发展。积极创办六安技师学院，支持六安一中兴建河西校区。深入开展爱国卫生运动，做好重大传染病防控工作。抓好**基层医药卫生体制综合改革，支持市人民医院西院区建设，**完成16个社区卫生服务中心（站）和11个乡镇卫生院、1178个村卫生室规范化建设。广泛开展群众性体育活动，实施219个农民体育健身工程。加快少数民族聚居地区经济社会发展，依法加强对宗教事务的管理。切实加强出生人口性别比综合治理，大力推进流动人口管理区域“一盘棋”，加快村级计划生育信息化建设和服务阵地建设。深入推进广播电视“村村通”等文化惠民工程，完成42个乡镇综合文化站和452个农家书屋建设任务。建成市博物馆，力争开工建设市文化馆、图书馆和新皖西大戏院。编制申报汉王陵区保护规划和大遗址公园项目，出台文化产业振兴规划。加强对外交流与合作，进一步做好外事、侨务、港澳和对台工作。继续巩固“全国双拥模范城”创建成果，大力支持驻地部队建设，进一步加强国防动员、国防后备力量和人民防空工作。支持工会、共青团、妇联、科协等人民团体独立自主开展工作。加快统计、地震、气象、档案、地方志等各项事业的发展。
　　推进生态文明建设。强化节能减排目标管理责任制，扎实开展节能环保专项行动，全面落实十项节能攻坚措施。精心组织节能减排工作方案实施，提高污水处理厂运行效益和管网覆盖率。严格执行新建项目“三同时”制度，抓好重点单位节能减排攻坚项目实施，完成“十一五”节能减排目标任务。认真实施造林绿化、退耕还林、水土保持等生态治理工程，抓好合六叶高速公路六安段绿色长廊建设，完成造林10万亩，治理水土流失面积80平方公里。抓好循环经济和农村清洁工程试点，大力推进资源节约和综合利用。深入开展省生态建设综合示范市建设和生态村创建工作，加快推进金寨县、舒城县创建国家级生态县步伐。
　　维护社会安全稳定。继续深入开展安全生产专项行动，加强体制机制和执法队伍建设。严格落实领导责任制和责任追究制，突出抓好重点行业、重点领域的安全生产。加强食品药品安全监管，保障公共饮食及用药安全。加强基层维稳工作，健全信访工作长效机制，强化矛盾排查化解，规范信访程序。推进政法体制经费保障机制改革，充实基层警力和一线力量，严厉打击各种严重刑事犯罪，加强农村治安整治，健全社会治安防控体系。推进民主法制建设，做好“五五”普法总结验收，扩大法律援助覆盖面。加强应急预案体系和体制机制建设，增强应对突发事件能力。
　　今年还有一项重要工作，就是编制完成“十二五”规划。我们将坚持以科学发展观为指导，充分发扬民主，凝聚各方智慧，深入研究我市经济社会发展中的重大战略问题，切实增强规划的科学性、前瞻性和指导性，加速推进“三大跨越”。
　　各位代表！完成既定任务，实现宏伟目标，需要全市上下共同努力，需要政府自身积极作为。我们必须密切联系群众，求真务实，艰苦奋斗，不断提高领导科学发展的能力和水平，努力建设法治政府、服务政府、责任政府和效能政府。
　　加强学习，不断解放思想。树立重视学习、坚持学习、终身学习的理念，坚持用科学理论武装头脑，用先进理念指导工作。要潜心研究和准确把握国家政策，结合六安实际用足用活，不断增强工作的预见性和创造性。把解放思想作为加快发展的金钥匙，把加快发展作为解放思想的试金石，凡是有利于又好又快发展的，坚定不移地去做；凡是不利于又好又快发展的，毫不迟疑地去改，真正把思想解放的成效体现在转变发展方式上，体现在破解发展难题上，体现在清除发展障碍上。
　　依法行政，从严管理队伍。健全领导干部学法制度，完善重大行政决策听取意见、听证、合法性审查、事后评价制度，注重依法决策、民主决策、科学决策。主动接受人大法律监督、政协民主监督以及新闻媒体、社会各界的舆论监督，高效办理人大代表议案、建议和政协委员提案。加大政务公开力度，重点推进公共权力大、公众关注度高、公益性强的部门和领域的信息公开。严格落实行政执法责任制和过错追究制，狠抓行政执法队伍建设，进一步规范行政执法行为，不断完善执法监督约束机制，确保严格执法、公正执法、文明执法。
　　强化服务，提高行政效能。积极推进市、县、乡镇政务服务中心标准化建设，加快建设市县一体化的网络综合办公系统和网上行政审批系统，推行行政审批网上办理。继续开展“发展环境暨政风行风万人评”活动，注重评议结果的运用。深入开展商业贿赂和损害群众利益不正之风专项治理活动，重点解决生态环境保护、食品药品质量、安全生产、教育、医疗等方面群众反映强烈的突出问题。严厉查处破坏经济发展环境的人和事，营造宽松和谐的干事创业氛围，使政府真正成为发展环境的创造者、社会公平的保障者、人民利益的维护者。
　　奋发有为，着力改进作风。始终保持昂扬的斗志、饱满的热情，大力发扬高效快捷、雷厉风行的工作作风。切实增强全市“一盘棋”的思想，杜绝不作为，摒弃慢作为，惩治乱作为，确保政令畅通。坚持深入基层，加强调查研究，做到情况在一线了解、问题在一线解决、形象在一线树立。积极运用信息化手段，大力精简会议和文件，改进会风文风，完善考评体系。政府各级领导干部都要身先士卒，抓好班子，带好队伍，形成抓落实、求突破的强大合力。
　　廉洁从政，树立良好形象。加强廉政文化建设，深入开展警示教育和岗位廉政教育。加大监察工作力度，切实加强对国有建设工程项目招投标、国土资源交易、国有产权交易、政府采购等方面的监督管理。发挥审计监督作用，加强对财政性资金投资项目的预决算审计和民生工程资金的跟踪审计，依法查处各种违纪违法案件。注重源头防治，突出重点领域和关键环节，不断完善防治腐败的体制机制。各级政府要带头压减一般性支出，勤俭办一切事业。政府全体工作人员尤其是领导干部必须带头廉洁从政，营造风清气正的政治生态。
　　各位代表！今天的六安，正处在充满活力、彰显魅力、积聚实力的重要发展阶段。宏伟的蓝图引领我们开拓进取，美好的未来激励我们奋勇拼搏。让我们在中共六安市委的坚强领导下，高举中国特色社会主义伟大旗帜，深入贯彻落实科学发展观，同心同德，扎实苦干，奋力谱写六安科学发展、跨越发展、和谐发展的新篇章！
